# Supplementary material for: Resolving the full spectrum of human genome variation using Linked-Reads
Source: Genome Res. 2019 Apr;29(4):635–45. doi: 10.1101/gr.234443.118 (PMC6442396; doi:10.1101/gr.234443.118)
Supplement: Supplemental Material [file supp_gr.234443.118_Supplemental_longranger-2.2.zip › longranger-2.2.tar.bz2!/lr2.2-public/lib/stan/cmdstan/stan/doc/api/html/math_2functions_2ibeta_8hpp_source.html]

Stan: src/stan/math/functions/ibeta.hpp Source File


|  |  |
| --- | --- |
|  | Stan  2.5.0  probability, sampling & optimization |


- Main Page
- Related Pages
- Namespaces
- Classes
- Files

- File List
- File Members

All Classes Namespaces Files Functions Variables Typedefs Enumerations Enumerator Friends Macros Pages

- src
- stan
- math
- functions

ibeta.hpp

Go to the documentation of this file.

1 #ifndef STAN\_\_MATH\_\_FUNCTIONS\_\_IBETA\_HPP

2 #define STAN\_\_MATH\_\_FUNCTIONS\_\_IBETA\_HPP

3

4 #include <boost/math/special\_functions/beta.hpp>

5 #include <stan/math/error\_handling/check\_not\_nan.hpp>

6

7 namespace stan {

8  namespace math {

9

23  inline double ibeta(const double a,

24  const double b,

25  const double x) {

26  check\_not\_nan("ibeta(%1%)", a, "a", static\_cast<double\*>(0));

27  check\_not\_nan("ibeta(%1%)", b, "b", static\_cast<double\*>(0));

28  check\_not\_nan("ibeta(%1%)", x, "x", static\_cast<double\*>(0));

29  return boost::math::ibeta(a, b, x);

30  }

31

32  }

33 }

34

35 #endif

check\_not\_nan.hpp

stan::math::ibeta

double ibeta(const double a, const double b, const double x)

The normalized incomplete beta function of a, b, and x.

**Definition:** ibeta.hpp:23

stan::math::check\_not\_nan

bool check\_not\_nan(const char \*function, const T\_y &y, const char \*name, T\_result \*result)

Checks if the variable y is nan.

**Definition:** check\_not\_nan.hpp:56


---

[ Stan Home Page ]

*© 2011–2014,
Stan Development Team.*
